# Association between fibrosis-related gene polymorphism and long-term allograft outcome in renal transplant recipients

**DOI:** 10.1186/s12920-023-01686-6

**Published:** 2023-10-23

**Authors:** Yu Yin, Han Zhang, Li Sun, Qianguang Han, Ming Zheng, Hao Chen, Shuang Fei, Ruoyun Tan, Xiaobing Ju, Zijie Wang, Min Gu

**Affiliations:** 1 Department of Urology, The First Affiliated Hospital of Nanjing Medical University, Nanjing, China; 2 Department of Urology, The Second Affiliated Hospital of Nanjing Medical University, Nanjing, China

**Keywords:** Kidney transplantation, Single-nucleotide polymorphisms (SNPs), Renal allograft fibrosis, Long-term outcome

## Abstract

**Background:**

Renal allograft fibrosis is one of characteristic causes of long-term renal function loss. The purpose of our study is to investigate the association between fibrosis-related genes single nucleotide polymorphism (SNPs) and kidney function in 5 years after kidney transplantation.

**Methods:**

A total of 143 recipients were eligible for screening with 5-year follow-up information and SNP sequencing information from blood samples were included in this study. Minor Allele Frequency (MAF) and Hardy–Weinberg Equilibrium (HWE) analysis was conducted to identify tagger single-nucleotide polymorphisms (SNPs) and haplotypes. SNPs associated with the fifth year chronic kidney disease (CKD) staging were screened by SPSS and the “SNPassoc” package in RStudio and used for subsequent prediction model construction.

**Results:**

A total of 275 renal transplant-related SNPs identified after target sequencing analysis. 64 Tagger SNPs were selected, and two SNPs (rs13969 and rs243849) were statistically significant for stage of CKD in 5 years. Finally, a model based on Gender, Age, rs1396, and rs243849 was constructed by multivariate linear regression analysis. Additionally, this model has a good performance in predicting uremia five years after kidney transplantation.

**Conclusion:**

Two SNPs (rs13969 and rs243849) were identified to be significantly associated with long-term renal allograft function. Based on this, a prediction model for long-term allograft function was established containing Gender, Age, rs1396, and rs243849. However, an independent cohort should be enrolled to validate the predicting performance.

**Supplementary Information:**

The online version contains supplementary material available at 10.1186/s12920-023-01686-6.

## Introduction

End-stage renal disease happens as the final and most severe stage of chronic kidney disease (CKD), with poor prognosis and high medical cost. Although transplantation of kidneys is the preferred treatment option for most end-stage renal diseases [1], the failure rate of kidney allografts remains high after a long period of time, with approximately 3% individuals who return to dialysis every year [2]. In various types of chronic kidney disease, renal fibrosis is a common pathological feature, regrading as a progressive process indicator in the diagnosis and evaluation of allograft failure [3, 4].

Fibrosis of the transplanted kidney clearly requires the involvement and interaction of various types of cells, including the activation of mesangial and fibroblast cells, the epithelial-mesenchymal transition (EMT) in tubular epithelium, the infiltration of monocyte/macrophage and T-cell as well as cell apoptosis [5]. In recent studies, it has been demonstrated that genetic polymorphisms and epigenetic variations determine progression rates of kidney fibrosis [6]. In the previous studies, genetic polymorphisms of caveolin-1 has correlation with the outcome of kidney transplant and arterial stiffness of CKD [7, 8].

Various physiological and pathophysiological processes are regulated by genetic factors, delivering an orientation for Intervention of renal allograft fibrosis at early stage. The genetic variation known as single nucleotide polymorphism (SNP) have an impact on gene functions of progression of disease. Some common single-nucleotide polymorphisms (SNPs; rs6276, rs6277, and rs1800497) in the human decrease the dopamine D2 receptor gene which have a correlation with the inflammation and fibrosis in human renal [9]. Jason et al. demonstrated the CAV1 rs4730751 SNP significantly contributed to allograft failure with a cohort of 697 people [7]. Fibrosis of transplanted kidney is an important factor that affects long-term renal function and even leads to the loss of transplanted kidney. Therefore, this study started from SNPs of fibrosis-related genes such as TGF-β, TGFBR, SMAD, MMP, TNF-α, OPN, CAV1/2, and screened SNPs related to long-term renal function.

In the process of fibrosis, diverse cytokines are secreted, for instance, TGF-β and its ligand TGFBR bind to activate transcription factors of the SMAD family, which can regulate the expression of proteins in cells and affect a series of kidney fibrosis processes, including Tubular Atrophy, EMT [10]. Based on previous research by our center, TNF-α also contributes to the progression of fibrosis by promoting EMT [11]. TGF-β also appears to be a pathway that integrates the effects of multiple other factors to promote fibrosis formation, such as the degradation of caveolin(CAV)-mediated TGF-β type I receptor internalization [12], as well as the infiltration of macrophage (Mø) which is one of the sources of TGF-β. Macrophage (Mø) infiltrate the interstitium by complex recruitment mechanisms which may depend in part on osteopontin(OPN) expression [13]. With the progression of fibrosis, a large amount of matrix is deposited in the kidney tissue. Previous studies have shown that the proteolytic network composed of matrix metalloproteinase (MMP) is abnormal, resulting in the defective function of degrading matrix proteins [5].

The research we conducted is divided into two parts. Firstly, we follow up the patients in the cohort over time to obtain clinical information and determine the long-term trend in renal function. Then, comparative research of 274 SNPs of 7 fibrosis-related gene was performed in kidney transplant recipients with next-generation sequencing (NGS) application. Lastly, we formulate a signature of SNPs to predict prognosis of kidney allografts.

## Material and methods

### Study design

This was a retrospective case–control study which contains 143 participants with available follow-up information during 5-years-term periods. The participants underwent kidney transplantation surgery between 1st February 2010 and 1st December 2015, at the First Affiliated Hospital with Nanjing Medical University. The included recipients met the following inclusion criteria: (1) recipients who were more than 18 years or less than 60 years, (2) recipients who either experienced stable serum creatinine levels (< 120 μmol/ L; fluctuation < 20%) for at least 3 months or were diagnosed with chronic allograft dysfunction (CAD) by laboratory and pathological examinations, and (3) recipients with follow-up for more than 5 years after kidney transplantation. The exclusion criteria included are as follows: (1) recipients who did not meet the inclusion criteria; (2) recipients with severe heart, liver, or lung disease or chronic viral infections; and (3) recipients diagnosed with pregnancy and lactation.

We followed up the clinical manifestation such as eGFR, urea nitrogen, and urine protein levels of patients as endpoint of allograft function at 1, 3, and 5 years. The eGFR calculated by modified glomerular filtration rate estimating equation for Chinese patients [14], is used in subsequent data analysis. The other demographic data of recipients, such as age, sex, and panel reactive antibodies (PRAs) at the time of kidney transplantation, and incidence of Acute Rejection (AR) episodes, Delayed Graft Dysfunction (DGF), and usage of RAPA, and immunosuppressive protocols, were reviewed from the medical records.

### Immunosuppressive protocols

Basiliximab or antihuman thymocyte immunoglobulin was used as induction therapy at kidney transplantation. In all participants, calcineurin inhibitors such as tacrolimus or cyclosporin A were administered as immunosuppressive regimens during the maintenance period. Detail immunosuppression protocols were elaborated in this previous paper [15]. The dosage of immune suppressants was adjusted according to the serum creatinine level and drug concentration.

### Sample collection, preparation, and sequencing

Our previous study described the detailed procedures of sample collection and the TS steps [15]. Peripheral blood samples (2 mL) were collected from each recipient included in our study and the DNA was extracted and concentrated, then the integrity of gDNA was tested by agarose gel electrophoresis. Then, the regions of interest were hybrided selectively to the gDNA samples, which further fragmented into pieces and followed by end repair, dA tailing, and sequencing adaptor ligation. The adapter-ligated DNA was amplified using polymerase chain reaction (PCR), and then quantitatively analyzed. Sequencing data were analyzed according to the human reference sequence UCSC hg19 assembly (NCBI build 37.2). Eventually, the FASTQ files were generated and further annotated into TS data containing SNPs, which were analyzed in our study.

### Statistical analysis

Unless otherwise specified, data are presented as mean + Standard Deviation (SD). The Hardy–Weinberg equilibrium (HWE), Minor Allele Frequency (MAF) and the linkage disequilibrium (LD) blocks were calculated using the Haploview version 4.2 software (Broad Institute, Cambridge, MA, USA). Genetic variants with MAF < 0.05 and/or HWE less than Adjusted-*P* value will be excluded for further analysis. We used one-way analysis of variance (ANOVA) and chi-square test to compare the clinical variables in comparison with 3 groups sectionalized by CKD stages. Ordinal logistic regression analysis was used to select candidate clinical variables, which was considered as confounders in the following covariance analysis to select Tagger SNP related with long-term renal function, by using SPSS 26.0 software (SPSS Inc., Chicago, IL, USA). A genotype association analysis of tagger SNPs was performed using the dominant model (minor allele homozygotes plus heterozygotes vs. major allele homozygotes), recessive model (minor allele homozygotes vs. heterozygotes plus major homozygotes), additive model (major homozygotes vs. heterozygotes vs. minor homozygotes), HET model (major homozygotes vs. heterozygotes) and HOM model (major homozygotes vs. minor homozygotes) by the RStudio version 4.0.5 software (Boston, MA, USA) implemented by the “SNPassoc” package version 2.0–2. Significant SNPs with long-term renal function were selected by taking the intersection of the results calculated by SPSS and “SNPassoc” package. Furthermore, general linear models (GLMs) were used to test associations between eGFR in the fifth years after the transplant and genotypes, by using SPSS 26.0 software (SPSS Inc., Chicago, IL, USA). *P* < 0.05 was considered statistically significant and the adjustment of *P*-value by the number of genetic variants was performed in LD and variants association analysis.

## Result

### Baseline characteristics of participants

**Fig. 1:**
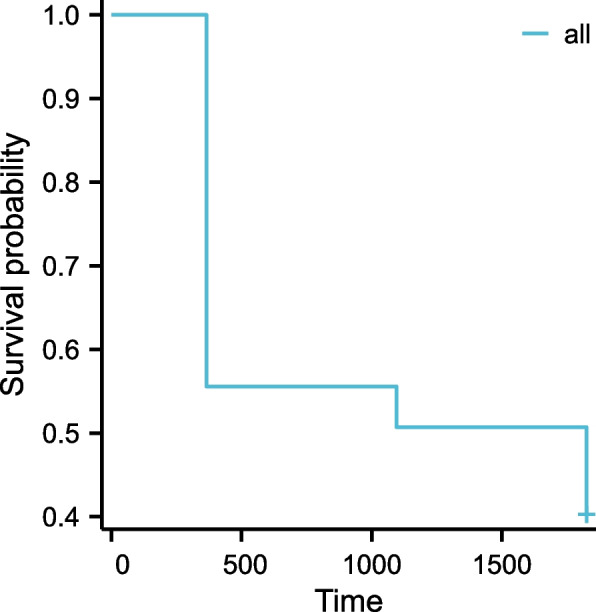
Kaplan–Meier survival curves showing the incidence of abnormal eGFR

In total, 143 recipients (including 38 women) were enrolled in this retrospective cohort study. In this study, the mean age of the patients was 36.87 ± 9.71 years, with a range of 10 to 59 years. Before kidney transplantation, none of the patients was detected with PRA. Primary kidney transplantation was performed on all recipients. Other baseline characteristics of recipients, including recipient weight, the presence of DGF, and incidence of AR before kidney transplant, and immunosuppressive protocols are presented in Table 1. The clinical manifestations of allografts are listed in Table 2, including GFR, urea nitrogen, and urine protein levels at 1, 3, and 5 years after surgery. Over the five years, the mean overall eGFR decreased from 97.49 ± 38.79 ml/min/1.73m^2^ in year 1 to 86.89 ± 41.13 ml/min/1.73m^2^ in year 5. By year 5, the proportion of patients with stage CKD1 had decreased from 55.2% to 43.4%. As a result, kidney function declines over time. A Kaplan–Meier (K-M) survival curve was used to demonstrate the overall renal function changes in the cohort over a 5-year period in Fig. 1. Comparing clinical variables in different CDK stages, we found statistically significant differences in age and gender (*P* > 0.05) in Table 3 (Age: *P* = 0.01, Gender: *P* = 0.009).

**Table 1:** Basic characteristic of recipients included in the cohort

**Variables**	**Value**
n	143
Age(years, mean ± SD)	36.87 ± 9.71
Gender,n(%)	
Male	105(73.4)
Female	38(26.6)
Weight(kg,mean ± SD)	60.55 ± 8.65
Duration after renal transplant	2695.10 ± 461.49
PRA before renal transplant (%)	0
Primary/secondary renal transplant	143/0
Incidence of DGF episodes, n (%)	51(35.7)
Incidence of AR episodes, n (%)	54(37.8)
Usage of RAPA, n (%)	17(11.9)
ISD protocol,n(%)	
CsA	54(37.8)
FK506	89(62.2)

*SD* Standard Deviations, *PRA* Panel Reactive antibodies, *DGF* Delayed Graft Function, *AR* Acute Rejection, *RAPA* Rapamycin, *ISD* Immunosuppressant Drug, *CsA* Ciclosporin A, *FK506* Tacrolimus

**Table 2:** The clinical manifestations of allografts

**Variables**	**1-year: allograft function**	**3-year: allograft function**	**5-year: allograft function**
BUN(mmol/L,mean ± SD)	7.49 ± 3.52	7.35 ± 7.37	8.80 ± 6.78
Scr(umol/L,mean ± SD)	105.60 ± 42.39	113.14 ± 54.13	136.83 ± 103.04
eGFR(ml/min/1.73m2,mean ± SD)	97.49 ± 38.79	92.31 ± 36.35	86.89 ± 41.13
Upro,n(%)			
-	128(88.8)	115(80.4)	107(74.8)
+	10(7.0)	18(12.6)	22(15.4)
+ +	2(1.4)	8(5.6)	10(7.0)
+ + +	3(2.1)	2(1.4)	2(1.4)
CKD,n(%)			
1	79(55.2)	72(50.3)	62(43.4)
2	38(26.6)	42(29.4)	38(26.6)
3	25(17.5)	26(18.2)	30(21.0)
4	0	2(1.4)	8(5.6)
5	0	1(0.7)	5(3.5)

*BUN* Blood Urea Nitrogen, *Scr* Serum Creatinine, *eGFR* estimated Glomerular Filtration Rate, *Upro* Urine Protein, *CKD* Chronic Kidney Diseases

**Table 3:** The comparison of clinical variables in different CKD stages

**Clinical variables**	**CKD1**	**CKD2-3**	**CKD4-5**	***P***
BUN(mmol/L,mean ± SD)	5.71 ± 1.50	8.46 ± 3.53	25.29 ± 10.82	> 0.001
Scr(umol/L,mean ± SD)	81.16 ± 13.79	132.73 ± 34.41	423.69 ± 113.16	> 0.001
Upro,n(%)				> 0.001
-	57	49	1	
+	4	12	6	
+ +	1	5	4	
+ + +	0	1	1	
+ + + +	0	1	1	
eGFR(ml/min/1.73m2,mean ± SD)	124.75 ± 26.69	65.49 ± 14.84	18.16 ± 6.68	> 0.001
Age(years, mean ± SD)	34.45 ± 9.328	38.01 ± 9.40	42.38 ± 10.47	0.01
Weight (kg,mean ± SD)	61.71 ± 9.39	59.10 ± 7.99	59.88 ± 7.31	0.107
Gender,n(%)				0.009
Male	55	41	9	
Female	7	27	4	
Duration after renal transplant(days, mean ± SD)	2661.53 ± 406.45	2711.21 ± 499.79	2770.92 ± 521.74	0.686
Incidence of DGF episodes, n (%)	26	21	4	0.391
Incidence of AR episodes, n (%)	22	27	5	0.883
Usage of RAPA, n (%)	8	8	1	0.869
ISD protocol,n(%)				0.634
CsA	26	23	5	
FK506	36	45	8	

### Tagger-SNP selection

Further analysis of SNPs was conducted for 7 genes related to fibrosis (Table S1). In Table S2, the genetic information for all detected SNPs is presented. There were 95 SNPs (Table S3) with MAF > 0.05 and HWE > 0.05 that remained for further analysis after HWE and MAF were calculated. In addition, LD analysis was carried out among all the SNPs to identify the tagger SNPs (Supplementary Figure 1). Finally, we obtained 64 Tagger SNPs (Table S4) for further association analysis.

### Associations of fibrosis related SNPs and eGFR

After Ordinal logistic regression for detecting the potential impact of confounding clinical variables on the stage of CKD, a total of three statistically significant confounding clinical variables were found, including age gender and incidence of AR episodes (age: OR = 1.061, *P* = 0.001; gender: OR = 2.365, *P* = 0.033; incidence of AR episode: OR = 0.437, *P* = 0.034; Table 4). After an analysis of covariance (ANCOVA) was performed to control for confounding factors, a total of six tagger SNPs were summarized to be statistically significant depending on different stages of CKD. An analysis of multiple inheritance models (dominant, recessive, additive, HET and HOM) was conducted to narrow down the candidate SNPs by applying Bonferroni correction (adjusted *P* value = 0.05). It was concluded that rs13969 and rs243849 were statistically significant for stage of CKD. Finally, we get rs13969 and rs243849 by taking the intersection between the ANCOVA result of SPSS and the multiple inheritance models result of “SNPassoc” package in RStudio (Table 5).

**Table 4:** Influence of confounding factors on the outcomes of proteinuria by a logistic regression model in this cohort

**Variables**	**β**	**OR**	**OR95%CI**	***P* value**
Age(years, mean ± SD)	0.060	1.061	1.026 ~ 1.098	0.001
Weight (kg,mea ± SD)	-0.037	0.964	0.922 ~ 1.008	0.103
Gender,n(%)	0.861	2.365	1.072 ~ 5.216	0.033
Incidence of DGF episodes, n (%)	0.412	1.509	0.756 ~ 3.015	0.244
Incidence of AR episodes, n (%)	-0.828	0.437	0.203 ~ 0.941	0.034
Usage of RAPA, n (%)	0.181	1.198	0.403 ~ 3.567	0.745
ISD protocol,n(%)	-0.251	0.778	0.374 ~ 1.617	0.501

**Table 5:** Results of multivariable linear regression analysis on the eGFR at year 5 in real transplant recipients

	**coef**	**t**	***P***
Gender	0.208	2.426	0.017
Age	-0.259	-3.341	0.001
rs13969	-0.198	-2.588	0.011
rs243849	-0.186	-2.422	0.017

### The construction of the prediction model

**Fig. 2:**
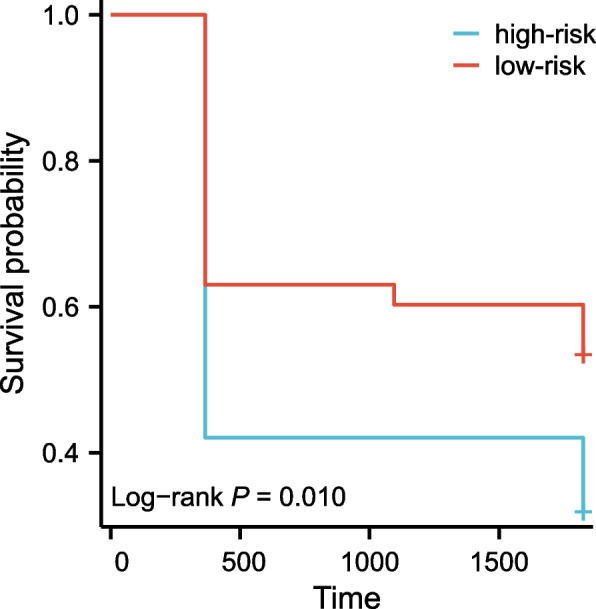
Kaplan–Meier survival curves in the high-risk group and low-risk group

Multivariate linear regression analysis was performed on 2 candidate SNP and clinical baseline characteristics. Finally, 4 Variables were identified and their risk-correlation coefficients were calculated to determine the prognosis of patients after kidney transplantation (Table 5). The risk score was calculated as follows: riskScore = Gender*0.208 + Age*-0.259 + rs13969*-0.198 + rs243849*-0.186. The risk score of each case in this group was calculated, and all cases were classified into the high-risk group (71 patients) and the low-risk group (72 patients) based on the median risk score of -16.94 (Table S5). Then, the Kaplan–Meier curves suggested that the constructed risk characteristics still had good predictive power in the stage of CKD (Fig. 2).

## Discussion

In this study, MMP9 rs13969 and MMP2 rs243849 were significantly correlated with eGFR of renal allograft. Previous research has linked MMP disorders with both acute and chronic renal pathophysiology, including acute kidney injury (AKI), glomerulosclerosis/renal tubulointerstitial fibrosis, diabetic nephropathy, polycystic nephropathy, and renal carcinoma [16]. The serum levels of MMP-2 and TIMP-2 are elevated in chronic kidney disease patients [17]. Similarity to our results, it has been shown that polymorphisms in MMP-2 and -9 are associated with improved allograft survival [18].

For our study, we included 143 recipients who met our exclusion criteria and had full 5-year follow-up information. The Modified glomerular filtration rate estimating equation, which is more suitable for Chinese people, was used to estimate the eGFR value of patients through the clinical information of patients, for the subsequent CKD staging of kidney transplantation patients [14]. As confounding factors in subsequent analyses, age, gender, and AR episodes were determined using ANOVA and chi-square tests in SPSS. For selecting tagger SNPS related to eGFR in the 5th year, we used covariance analysis in SPSS and multiple inheritance models in the “SNPassoc” package in R, then taking the intersection of the two results. Finally, based on two candidate SNP and clinical baseline characteristics, we used multivariate linear regression analysis to construct a long-term riskScore prediction model. There is a significant difference between low-risk and high-risk groups distinguished by their riskScore, demonstrating good predictive power in the stage of chronic kidney disease. The variable on which we constructed our risk model is the patient’s innately determined SNPs, thus allowing us to identify patients at higher risk in the early transplantation process and personalise their management, for example, by increasing the frequency of postoperative review of the patient, and detecting the development of post-transplantation adverse events at an early stage to reduce their adverse effects on the patient. Secondly the SNPs we identified may serve as potential targets for future antifibrotic drug development. Finally, the SNPs we screened for that are associated with long-term transplanted kidney function could also be used in subsequent bioinformatic studies, for example to guide the selection of instrumental variables in Mendelian randomisation studies.

Fibrosis of the transplant kidney is characterized by pathological manifestations such as glomerular sclerosis, renal tubule atrophy, interstitial fibrosis of the renal tubule, and inflammatory cell infiltration, capillary remodeling and other pathophysiological features. It is now accepted that kidney fibrosis is formed as a result of the body’s healing response [19]. After initial damage, inflammatory mononuclear macrophages infiltrate the renal interstitium and produce molecules that encourage fibrosis, such as reactive oxygen species (ROS) and inflammatory cytokine. These fibrosis-promoting cytokines further promote mesangial and fibroblast activation, tubular epithelial to mesenchymal cell transition (EMT) [20], mononuclear/macrophage, and T cell infiltration. The final result is a large amount of extracellular matrix (ECM) deposition, leading to the destruction of the normal structure of the kidney and the loss of kidney function [5].

MMPs belong to a 23-member family of endopeptidase, which contain zinc, are dependent on calcium, and can degrade and remodel the proteins that form the extracellular matrix(ECM) [21], whose excessive accumulation is the main pathological mechanism of renal fibrosis [22]. Therefore, the proteolytic network composed of MMPs has been regarded as an important factor in alleviating renal fibrosis after injury. As members of MMP family, MMP-2 and -9 have a series of fibronectin repeats in catalytic structural domain, and have the ability to cleave denatured collagen (gelatin) as well as type IV collagen in basement membranes [23]. At the same time, it is the accumulation of type I, III, and IV collagen in the glomeruli, interstitial tissues, and blood vessels that leads to progressive renal fibrosis [24]. MMP-2 and MMP-9 can also stimulate the creation of fibrosis by activating TGF-β1 and its associated fibrosis pathways [25]. The accumulation of ECM in renal fibrosis comes not only from fibroblasts in the renal tubule interstitium and perivascular, but also from myoblasts, which is the result of EMT [26, 27]. The degradation ability of MMP-2 and -9 may adversely affect the integrity of renal parenchyma, inducing EMT [28], leading to spontaneous tubular atrophy consistent, resulting in the progression of CKD [23]. According to Ye els, MMP9 plays a key role in EMT induced by Notch signaling, which regulate kidney development [29]. As demonstration by Sunfa els, MMP2 is required for EMT induced by TGF-1, for MMP2 alone is sufficient to initiate EMT in the absence of TGF- 1 [30].

However, as the common final outcome of CKD, fibrosis in transplanted kidneys is not impacted only by accumulation degradation of ECM, but also by inflammation and vascular remodeling. Inflammatory cells are recruited by MMPs in the early stages of inflammation. Collagen fragments created by MMP-9, could recruit neutrophils and stimulate them to release more MMP-9 [31]. Meanwhile, MMP-9 mediates dendritic cell (DC) migratory [32]. In the stage of immune rejection, T cell alloreactivity and inflammatory cytokine release are reduced in MMP-2 null mice, while they are increased in MMP-9 null mice [33]. MMPs also play an important role in modulating vascular remodeling. Microvascular losses are reduced in MMP-9 null mice after ischemia [34]. Collagens, MMP-mediated degradation generate angiostatin, which is antiangiogenic, increasing in ischemic injury [35]. Relaxation of vessels can be improved by MMP inhibitors [36]. These renal vasculature changes caused by MMP could portend worsened renal prognosis and increased vulnerability to future injuries.

Even so, there are still limitations in our entire research process. First of all, the description of the mechanism of action described in this article is not specific enough. Secondly, even though our predictive model is equipped with satisfactory performance in our dataset, the sample size in our dataset is relatively small. However, the SNP data in our study are from peripheral blood, thus the predictive model can be applied to determine the likelihood of eGFR through timely blood tests, which suggests our model has an outstanding classification performance. Our predictive model needs to be investigated further in clinical work.

## Conclusion

In our study, MMP9 rs13969 and MMP2 rs243849 was found to be statistically associated with the eGFR in the 5 years after renal transplantation. Moreover, a prediction model for long-term allograft function containing gender, age, rs1396, and rs243849 was established. However, an independent cohort should be enrolled to further validate the prediction efficacy of this model in the prognosis of long-term allograft function.

### Supplementary Information

**Additional file 1: Supplementary Figure 1.** Linkage disequilibrium results of detecting tagger SNPs in the fibrosis-related genes.

**Additional file 2: Table S1.** The genes related to fibrosis.

**Additional file 3: Table S2.** Genetic information on SNPs of fibrosis-related genes.

**Additional file 4: Table S3.** Results of HWE and MAF analysis.

**Additional file 5: Table S4.** Results of LD analysis.

**Additional file 6: Table S5.** The risk score of high-risk group and low-risk group.

## Data Availability

Genetic expression files are posted on the Sequence Read Archive (SRA) database (https://www.ncbi.nlm.nih.gov/sra; SRP133091).
