# Supplementary figures and images for: Association between fibrosis-related gene polymorphism and long-term allograft outcome in renal transplant recipients
Source: BMC Med Genomics. 2023 Oct 23;16:255. doi: 10.1186/s12920-023-01686-6 (PMC10591404; doi:10.1186/s12920-023-01686-6)

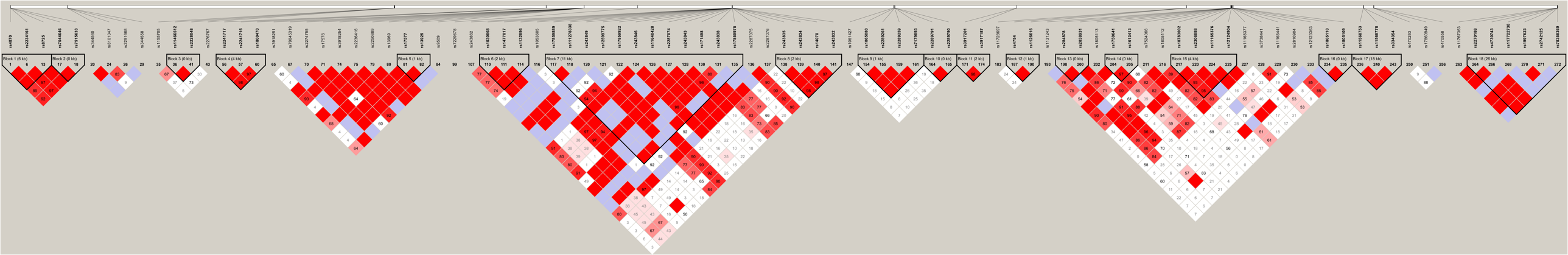

Supplement: Supplementary file 1 — Additional file 1: Supplementary Figure 1. Linkage disequilibrium results of detecting tagger SNPs in the fibrosis-related genes. [file 12920_2023_1686_MOESM1_ESM.pdf]
